# The Impact of the Rate Prior on Bayesian Estimation of Divergence Times with Multiple Loci

**DOI:** 10.1093/sysbio/syu020

**Published:** 2014-03-21

**Authors:** Mario Dos Reis, Tianqi Zhu, Ziheng Yang

**Affiliations:** ^1^Department of Genetics, Evolution and Environment, University College London, Darwin Building, Gower Street, London WC1E 6BT, UK;; ^2^Beijing Institute of Genomics, Chinese Academy of Sciences, Beijing 100101, China

## Abstract

Bayesian methods provide a powerful way to estimate species divergence times by combining information from molecular sequences with information from the fossil record. With the explosive increase of genomic data, divergence time estimation increasingly uses data of multiple loci (genes or site partitions). Widely used computer programs to estimate divergence times use independent and identically distributed (i.i.d.) priors on the substitution rates for different loci. The i.i.d. prior is problematic. As the number of loci (*L*) increases, the prior variance of the average rate across all loci goes to zero at the rate 1/*L*. As a consequence, the rate prior dominates posterior time estimates when many loci are analyzed, and if the rate prior is misspecified, the estimated divergence times will converge to wrong values with very narrow credibility intervals. Here we develop a new prior on the locus rates based on the Dirichlet distribution that corrects the problematic behavior of the i.i.d. prior. We use computer simulation and real data analysis to highlight the differences between the old and new priors. For a dataset for six primate species, we show that with the old i.i.d. prior, if the prior rate is too high (or too low), the estimated divergence times are too young (or too old), outside the bounds imposed by the fossil calibrations. In contrast, with the new Dirichlet prior, posterior time estimates are insensitive to the rate prior and are compatible with the fossil calibrations. We re-analyzed a phylogenomic data set of 36 mammal species and show that using many fossil calibrations can alleviate the adverse impact of a misspecified rate prior to some extent. We recommend the use of the new Dirichlet prior in Bayesian divergence time estimation. [Bayesian inference, divergence time, relaxed clock, rate prior, partition analysis.]

Bayesian estimation of species divergence times from molecular sequence data is an unconventional statistical estimation problem. Molecular sequences provide information about the distances between species in a phylogeny, but not about the ages of clades or the molecular evolutionary rate, so that the model is not fully identifiable. Usually information from the fossil record is used to calibrate molecular trees and estimate clade ages (Thorne et al. 1998; Drummond et al. 2006; Yang and Rannala 2006; Lepage et al. 2007). Yang and Rannala (2006) and Rannala and Yang (2007) have shown that as the number of loci and the number of sites in molecular data increase, the uncertainty in posterior time estimates (measured by, e.g., the posterior variance) does not go to zero, but converges to a limiting value imposed by the uncertainty in the fossil calibrations. Therefore, although the uncertainty in time estimates cannot be eliminated, using many loci is desirable to reduce the posterior variance of time estimates as much as possible. With the growth of molecular sequence data, divergence time estimation will increasingly be conducted using multiple loci (or site partitions).

Current Bayesian divergence time estimation programs such as MCMCtree (Yang 2007), BEAST (Drummond et al. 2012), MrBayes (Ronquist et al. 2012), etc., allow different loci (site partitions) to have different overall rates, but use i.i.d. priors for the locus rates (the substitution rate per site valid for the locus). That is, the locus rates are assumed to be independent and identically distributed random variables from a common distribution such as the gamma or log-normal. However, this prior suffers from two major problems. First, with this prior, the prior uncertainty about the average rate over loci disappears when the number of loci increases. Suppose the locus rate, that is, the average substitution rate among the branches of the phylogeny at locus *i*, is μ_*i*_, for *i* = 1,…,*L*, where *L* is the number of loci. If the μ_*i*_ are i.i.d. with mean *m* and variance *v*, the mean rate across all loci, 
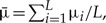
 will have mean *m* and variance *v*/*L*, so that as the number of loci goes to infinity (*L* → ∞), the prior variance of 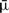 goes to zero (*v*/*L* → 0). Thus the prior makes increasingly strong and possibly implausible statement about the average rate 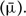 Second, the rate prior may exert an unexpectedly strong influence on the posterior time estimates. From the infinite-sites theory of Yang and Rannala (2006) and Rannala and Yang (2007), forcing the prior variance of 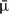 to zero will cause the posterior estimates of divergence times to approach point values (with zero variance) with the increase in the amount of sequence data (the number of sites at each locus and the number of loci). If the prior on locus rates is misspecified, posterior time estimates will be affected as well. For example if the prior rate is too high (or too low), the estimated times will be too young (or too old).

Those problems are general and affect posterior time estimation under all three commonly used clock models: the strict clock, the independent-rates model (Drummond et al. 2006; Rannala and Yang 2007), and the correlated-rates model (Thorne et al. 1998; Rannala and Yang 2007), as long as we use multiple loci and independent priors on locus rates (see also Huelsenbeck et al. 2000; Lepage et al. 2007; Heath et al. 2012 for more clock models). Figure 1 illustrates the problems, using the divergence between the human and chimpanzee as an example. The fossil record indicates that the human and chimpanzee diverged around 5.7 to 10 Ma (Benton et al. 2009) and molecular studies indicate a mean rate at the 3rd codon positions of protein-coding genes of around 10^−9^ site^−1^ year^−1^ (e.g., dos Reis and Yang 2013). When we use 300 loci (3rd codon positions in 300 genes) and a prior mean rate that is too high (10^−8^ site^−1^ year^−1^), the estimated divergence time is too young, at 3.3 Ma (with 95% credibility interval: 2.9–3.6), whereas for a prior mean rate that is too low (10^−10^ site^−1^ year^−1^) the estimated time is too old, at 26.4 Ma (24.7–28.2). In both cases the estimated times are outside the fossil calibration bounds. In contrast to a typical Bayesian analysis, in which the impact of the prior becomes less important when more data are available, here the prior becomes more influential when more data (more loci) are analyzed.

**Figure 1.**
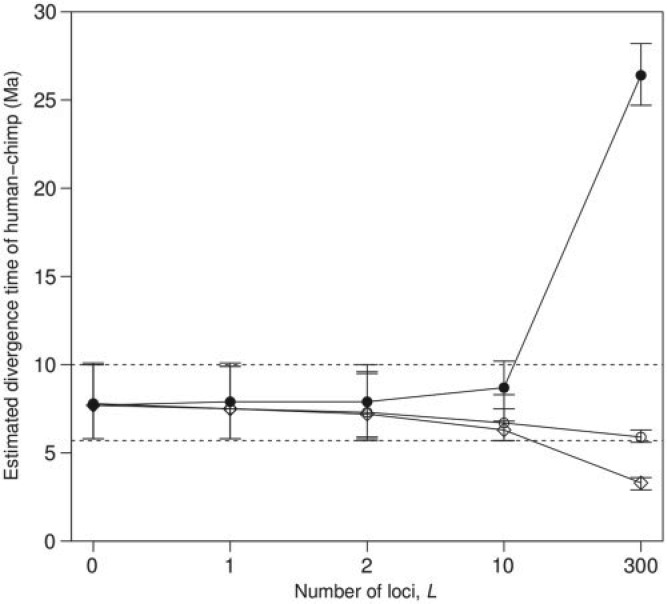
Posterior estimates of the human–chimpanzee divergence time under the i.i.d. prior for locus rates as the number of loci (*L*) increases and when the rate prior is misspecified. Genes were sampled randomly (without replacement) from six primate genomes, and then analyzed with the program MCMCtree. Fossil-based calibrations are placed on all five nodes in the tree, including the constraint of between 5.7 and 10 Ma for the human–chimpanzee divergence (see Fig. 4). Three priors on locus rates were used: (1) A fast rate, μ_*i*_∼*G*(2,2) (diamonds); (2) A medium rate μ_*i*_∼*G*(2,20) (empty circles); and (3) A slow rate, μ_*i*_∼*G*(2,200) (black circles). These priors have means 1, 0.1, and 0.01 respectively, in substitutions per site per 10^8^ years. When the prior rate is too fast, the estimated time becomes younger as *L* is increased. On the other hand, when the prior rate is too slow, the time becomes older with increased *L*. In both cases, the posterior times are outside the fossil bounds (dashed lines) when *L* = 300 loci are used. For the medium rate, the time also becomes younger. The data and fossil calibrations are from dos Reis and Yang (2013). The data set is analyzed later in this article, where full details of the analysis are given. Estimates for other node ages are given in Table 3.

In this article we implement a new prior on rates for loci that is robust to rate prior misspecification and that does not produce overly precise time estimates with many loci. We use computer simulation and real data analysis to study the different effects of the old and new rate priors on divergence time estimation.

## Theory

### The i.i.d. Prior on Rates for Loci

Here we review the i.i.d. prior on rates for loci implemented in current Bayesian molecular clock dating programs, to introduce the notation and to illustrate the problems of the i.i.d. prior. Let the mean rate for locus *i* be μ_*i*_, with *i* = 1,2,…,*L*. Under the global clock model, μ_*i*_ is the rate for all branches at the locus. Under the independent-rates model (Drummond et al. 2006; Rannala and Yang 2007), μ_*i*_ is the mean of the common distribution for all branch rates; for example, the rates for branches in the tree at locus *i* may be i.i.d. variables from the log-normal or gamma distribution with mean μ_*i*_. Under the correlated-rates model (Thorne et al. 1998; Kishino et al. 2001; Rannala and Yang 2007), μ_*i*_ is the rate at the root of the tree at the locus, from which rates for other nodes or branches evolve according to a stochastic process such as the geometric Brownian motion.

The posterior distribution of times (**t**), branch rates (**r**), and locus rates μ = {μ_1_,…,μ_*L*_}, given the molecular data (*D*), is
(1)
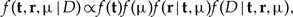

where *f*(**t**) is the prior on times, *f*(μ) is the prior on the *L* locus rates, *f*(**r**|**t**,μ) is the prior on branch rates, and *f*(*D*|**t**,**r**,μ) is the likelihood. The branch rates, **r**, are among loci and branches of the phylogeny. For example, if we analyze a phylogeny with 10 branches using 20 loci, we estimate 20 locus rates (μ) and 200 branch rates. If we assume that the rates among loci are independent, the prior on the locus rates is
(2)
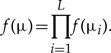


Multiplication of the independent densities together as in equation (2) leads to the problem that the prior (and thus the posterior) variance of the mean rate across loci goes to zero as *L* goes to infinity. Because times and rates are confounded (i.e., the likelihood function depends only on the product of times and rates), the informative rate prior has an undue and undesirable impact on the posterior distribution. Therefore, we propose a new prior on rates for loci.

### A New Prior on Rates for Loci

We implement a new prior on substitution rates for loci based on the Dirichlet distribution. This is similar to the Dirichlet prior of mutation rates among loci of Burgess and Yang (2008) for estimation of ancestral population sizes and to the compound-Dirichlet prior for branch lengths of Rannala et al. (Rannala et al. (2012, see also Zhang et al. 2012) for Bayesian phylogenetics. Our new prior is for the mean rates for the *L* loci, μ = {μ_1_,…,μ_*L*_}, and affects the implementations under all three clock models: the global clock, the independent-rates model, and the correlated-rates model.

We first assign a gamma prior on the mean rate 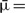

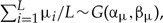
 with density
(3)
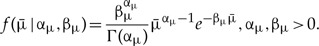

This has mean α_μ_/β_μ_ and variance 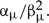 A small α_μ_, such as 1 or 2, means that the prior will be fairly diffuse about the mean rate over loci 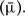 Next we partition the total rate 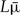 among the *L* loci using a Dirichlet distribution. In other words, the proportions 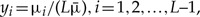 have a symmetrical Dirichlet distribution with concentration parameter α, with density
(4)
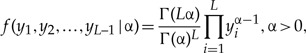

where *y*_*L*_ = 1−y_1_−y_2_−,…,−*y*_*L*_−1. A smaller α means greater variation in rates among loci. If α = 1, the distribution is called uniform Dirichlet, which is a multivariate generalization to the U(0, 1) distribution. By applying a variable transform 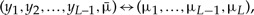 we obtain the joint distribution of the rates for the *L* loci as
(5)
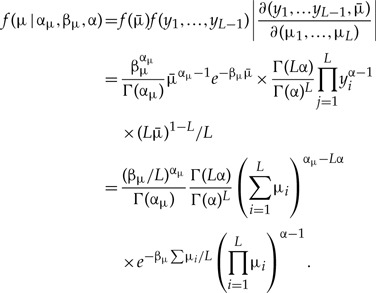

Note that for the special case α = α_μ_/*L*, equation 5 reduces to the joint density of *L* independent gamma variables: μ_*i*_∼*G*(α_μ_/*L*,β_μ_/*L*)

The marginal mean and marginal variance of μ_*i*_ are
(6)
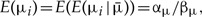

(7)
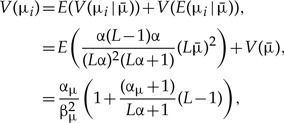

and the correlation between any pair μ_*i*_ and μ_*j*_ is
(8)
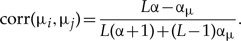

**Figure 2.**
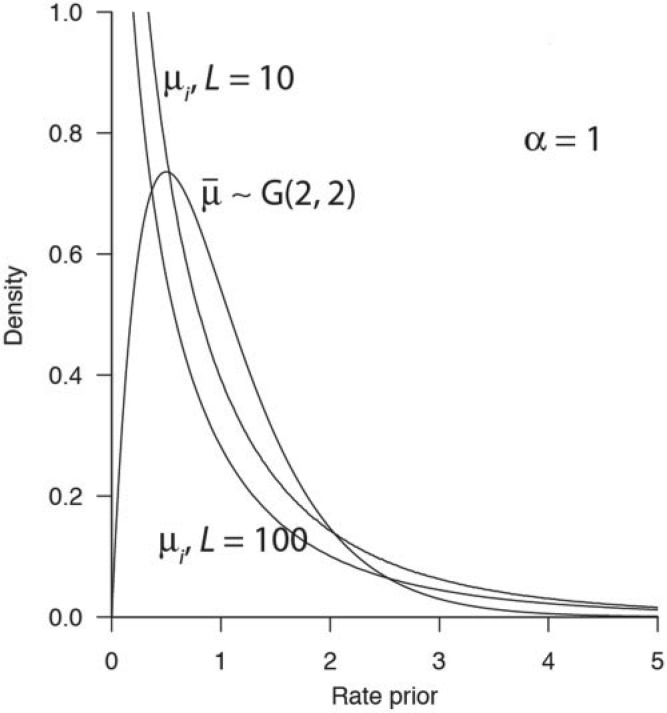
The marginal prior density *f*(μ_*i*_) implied by the new Dirichlet prior for locus rates. Given 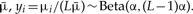 We simulated 10^7^ values of 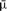 and *y*_*i*_, calculated 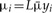 and plotted the kernel density estimate.

Figure 2 shows the marginal density *f*(μ_*i*_) implied by the new Dirichlet prior. If the parameters (α_μ_,β_μ_,α) are fixed and the number of loci (*L*) increases, the marginal density of μ_*i*_ becomes more diffuse, with a longer tail (and a larger variance).

In the relaxed-clock models, parameter 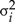 specify how variable the rate is among branches or how seriously violated the molecular clock is at locus *i* (e.g., Rannala and Yang 2007). For example, 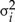 may be the variance of the log-rate in the log-normal distribution in the independent-rates model. In current Bayesian-dating programs, i.i.d. priors have been assigned to the variance parameters among loci, for example, 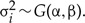 We also implement the Dirichlet prior for the locus-specific 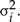 Our preliminary tests suggest that the prior on 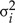 does not have such a dramatic impact on posterior time estimates as the prior on locus rates (μ_*i*_).

The new prior for μ_*i*_ and 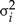 has been implemented in the program MCMCtree in the PAML package (Yang 2007) version 4.8. Our modification here affects only the calculation of the priors for μ_*i*_ and 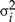 and the proposal steps to modify those parameters in the MCMC algorithm remain largely unchanged.

## Analysis of theTwo Species Case

### The Case of Finite Number of Loci and Infinite Number of Sites

We analyze the simple case of estimating the divergence time between two species under the strict clock, to examine the effects of the old i.i.d. prior and the new Dirichlet prior. First we consider data in which the number of sites at each locus is *N* = ∞ but the number of loci is finite. Because each locus is infinitely long, the molecular distances, *d*_*i*_ = 2*t*μ_*i*_, are known without error. This case is analyzed using the infinite-sites theory of Yang and Rannala (2006), which examines the asymptotic dynamics of posterior time estimates when the amount of sequence data goes to infinity (this should not be confused with the infinite-sites model in population genetics; see Kimura 1969, 1983). The posterior distribution of the time given the distances, **d** = (*d*_*i*_), under the i.i.d. rate prior is given by the infinite-sites theory (Yang and Rannala 2006, eq. 21) extended to *L* loci:
(9)
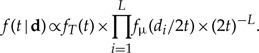


**Figure 3.**
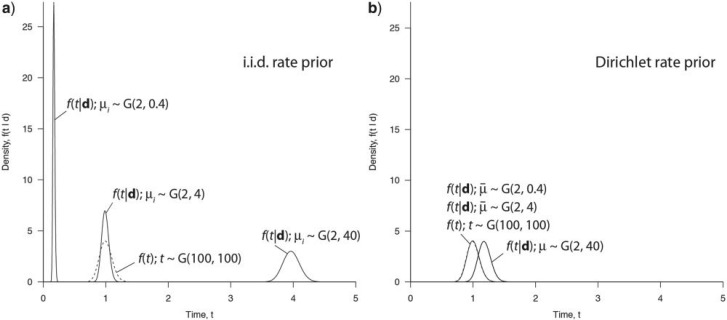
Posterior distribution of time *t* between two species, *f*(*t*∣**d**), under (A) The old i.i.d. prior and (B) The new Dirichlet prior. The true time is *t* = 1, and the true mean rate is 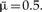 The data consist of 100 loci each of an infinite number of sites (*N* = ∞). When the data are analyzed, the prior on the divergence time is *t*∼*G*(100,100), shown as the dashed line. The three i.i.d. priors for locus rates are μ_*i*_∼*G*(2,0.4), μ_*i*_∼*G*(2,4), and μ_*i*_∼*G*(2,40). The three new Dirichlet priors for locus rates are 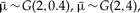 and 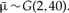

Consider the true time to be *t* = 1. If one time unit is 100 Myr, the true age will be 100 Ma. Suppose we sample 100 locus rates from a gamma distribution μ_*i*_∼*G*(2,4), with mean *E*(μ_*i*_) = 0.5, corresponding to 0.5 substitutions per site per 100 Myr, and set *d*_*i*_ = 2μ_*i*_*t*. The infinite-sites data at the 100 loci are then represented by the 100 *d*_*i*_ variables. These are analyzed using three locus rate priors: *G*(2,40), with mean 0.05 (slow); *G*(2,4), with mean 0.5 (good); and *G*(2,0.4), with mean 5 (fast). The time prior is *t*∼*G*(100,100), with mean 1, corresponding to a fossil calibration of 81–121 Ma (95% interval) for a true age of 100 Ma. The posterior distributions of time *t* (given by equation 9) under the three locus-rate priors are shown in Figure 3a. First, when the prior rate is good, the posterior density for time *t* is narrower than the prior density and located around the true time *t* = 1. Second, when the prior rate is ten times too fast, the posterior time density is very narrow and the posterior mean time is too young at 0.17. Finally, when the prior rate is ten times too slow, the posterior time density is wide and the posterior mean time is too old, at 3.97. Note that the posterior standard deviation of the time is proportional to the posterior mean time, or in other words the coefficient of variation (the standard deviation over the mean) is constant in the three cases, as predicted by the infinite-sites theory (Yang and Rannala 2006).

With the new Dirichlet prior, the posterior of time *t* given the distances is
(10)
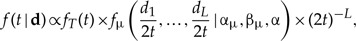

where *f*_μ_ is now given by equation (5). Consider the example above, except that this time the infinite data is analyzed with the new Dirichlet prior, and with three priors on 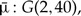 *G*(2,40), *G*(2,4) and *G*(2,0.4). The posterior distribution of *t* under the three locus-rate priors are shown in Figure 3b. In this case, the prior distribution of *t*, and the posterior distribution of *t* for the good and fast priors are all nearly identical, and centered around the true time *t* = 1. For the slow prior, the posterior distribution of *t* is shifted to the right (old ages) and centered around 1.16. Overall the posterior is much more robust to prior misspecification when the new Dirichlet prior is used than when the old i.i.d. prior is used.

### The Case of Finite Number of Loci and Finite Number of Sites

Next, we consider the case of finite data, with *N* = 1000 sites at each locus. Data are simulated and analyzed using the JC69 substitution model (Jukes and Cantor 1969). The true time is *t* = 1, and the true mean rate across loci is 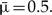 Suppose one time unit is 100 Myr. Then the true age of divergence is 100 Ma, and the true rate is 5 × 10^−9^ site^−1^ year^−1^. We simulate *L* = 1,2,10, and 100 loci, with 100 replicate data sets for each *L*. For locus *i*, we sample two rates for the two branches of the tree (*r*_*i*,1_ and *r*_*i*,2_) from the log-normal distribution: *r*∼*LN*(logμ_*i*_ − σ^2^/2,σ^2^) with σ^2^ = 0.1. The molecular distance between the two species for locus *i* is *d*_*i*_ = (*r*_*i*,1_ + *r*_*i*,2_)*t*. The program EVOLVER is then used to simulate the sequence alignments for each locus according to the value of *d*_*i*_.

The program MCMCtree is used to estimate the divergence time using the simulated alignments. The priors are the same as in the analysis of the inifinite-sites data, with *t*∼*G*(100,100) (with 95% interval 0.81–1.21). Using the old i.i.d. prior, we analyze each simulated data set three times, using three different locus rate priors: μ_*i*_∼*G*(2,0.4), μ_*i*_∼*G*(2,4) and μ_*i*_∼*G*(2,40). With the new Dirichlet prior, we analyze each data set three times, using three priors on the mean locus rate: 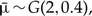

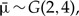
 and 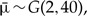 with α = 1. For each one of the six analyses, we calculate 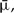 the average of μ_*i*_ over the *L* loci using the MCMC sample. We construct the posterior means and the 95% credibility interval (CI) of 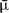 and *t*.

Tables 1 and 2 show the results of the simulation experiment. With the old i.i.d. prior on locus rates, the posterior estimate of time *t* is sensitive to the rate prior and the number of loci. As *L* becomes larger, *t* becomes too young if the prior rate is too fast, or too old if the prior rate is too slow (Table 1). Also, as *L* increases, the posterior of the mean rate 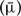 converges to wrong values. Consider for example the case of *L* = 100 loci. The good prior, μ_*i*_∼*G*(2,4), gives the posterior mean for *t* at 1.03 with 95% CI to be (0.917, 1.156). The posterior mean is close to the truth, but the intervals are too narrow. The fast-rate prior, μ_*i*_∼*G*(2,0.4), gives the posterior mean for *t* at 0.187 (0.157, 0.222), which is too young, and the posterior 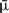 at 2.969 (2.496, 3.488), which is about six times too fast. With the slow-rate prior, μ_*i*_∼*G*(2,40), the posterior mean for *t* is too old, at 3.765 (3.502, 4.041), and the posterior 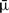 is too slow, at 0.119 (0.111, 0.128). In conclusion, for large *L* the posterior estimates of *t* and 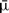 are too sensitive to rate prior misspecification.

**Table 1.** Posterior means and 95% CIs for the mean rate 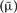 and the divergence time *t* between two species under the old i.i.d. prior

*L*	μ_*i*_∼	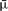	(95% CI)	*t*	(95% CI)
*L* = 0	G(2, 40)	0.05	(0.006, 0.139)	1	(0.813, 1.205)
	G(2, 4)	0.5	(0.06, 1.392)	–	–
	G(2, 0.4)	5.0	(0.606, 13.929)	–	–
*L* = 1	G(2, 40)	0.234	(0.114, 0.382)	1.073	(0.881, 1.284)
	G(2, 4)	0.527	(0.321, 0.82)	1.001	(0.816, 1.205)
	G(2, 0.4)	0.605	(0.357, 1.062)	0.982	(0.798, 1.186)
*L* = 2	G(2, 40)	0.23	(0.145, 0.328)	1.144	(0.948, 1.358)
	G(2, 4)	0.519	(0.360, 0.731)	1.001	(0.819, 1.203)
	G(2, 0.4)	0.627	(0.416, 0.972)	0.965	(0.783, 1.167)
*L* = 10	G(2, 40)	0.206	(0.172, 0.243)	1.624	(1.406, 1.857)
	G(2, 4)	0.521	(0.419, 0.641)	1.009	(0.837, 1.198)
	G(2, 0.4)	0.722	(0.548, 0.952)	0.829	(0.663, 1.014)
*L* = 100	G(2, 40)	0.119	(0.111, 0.128)	3.765	(3.502, 4.041)
	G(2, 4)	0.508	(0.453, 0.567)	1.031	(0.917, 1.156)
	G(2, 0.4)	2.969	(2.496, 3.488)	0.187	(0.157, 0.222)

Notes: *L* is the number of loci, with the results for *L* = 0 to be the prior. Each locus has *N* = 1,000 sites. The mean rate 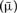 is calculated by averaging the locus rates from the MCMC sample. Note that the true mean rate is 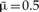 and the true time is *t* = 1.

**Table 2.** Posterior means and 95% CIs for the mean rate 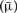 and the divergence time *t* between two species under the new Dirichlet prior

*L*	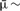	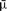	(95 %CI)	*t*	(95 %CI)
*L* = 0	G(2, 40)	0.05	(0.006, 0.139)	1	(0.813, 1.205)
	G(2, 4)	0.5	(0.060, 1.392)	–	–
	G(2, 0.4)	5.0	(0.606, 13.929)	–	–
*L* = 1	G(2, 40)	0.234	(0.114, 0.38)	1.074	(0.881, 1.284)
	G(2, 4)	0.52	(0.317, 0.811)	1.001	(0.816, 1.204)
	G(2, 0.4)	0.598	(0.353, 1.05)	0.983	(0.798, 1.186)
*L* = 2	G(2, 40)	0.318	(0.192, 0.453)	1.107	(0.915, 1.318)
	G(2, 4)	0.531	(0.349, 0.797)	1.001	(0.817, 1.205)
	G(2, 0.4)	0.573	(0.384, 0.899)	0.982	(0.798, 1.186)
*L* = 10	G(2, 40)	0.432	(0.324, 0.554)	1.152	(0.964, 1.358)
	G(2, 4)	0.531	(0.417, 0.682)	1.001	(0.817, 1.204)
	G(2, 0.4)	0.552	(0.429, 0.721)	0.982	(0.798, 1.186)
*L* = 100	G(2, 40)	0.443	(0.372, 0.525)	1.157	(0.970, 1.363)
	G(2, 4)	0.52	(0.426, 0.634)	1.001	(0.816, 1.204)
	G(2, 0.4)	0.527	(0.430, 0.645)	0.982	(0.798, 1.186)

Note: See note for Table 1.

The situation is quite different for the new Dirichlet prior. The posterior estimate of *t* is rather insensitive to *L* and to the rate prior (Table 2). Furthermore, the posterior of the mean rate is close to the true mean rate (0.5), even when the prior rate is either too fast or too slow (Table 2). For example, with *L* = 100, the posterior estimates of *t* are 1.157 (0.970, 1.363), 1.001 (0.816, 1.204), and 0.982 (0.798, 1.186), for the slow, good and fast rate priors respectively, all close to the true value *t* = 1. Similarly, with *L* = 100, the posterior estimates of 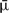 are 0.443 (0.372, 0.525), 0.520 (0.426, 0.634), and 0.527 (0.430, 0.645), for the slow, good and fast rate priors, all close to the true value 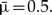


## Analysis of aSix-Species Primate Phylogeny

**Figure 4.**
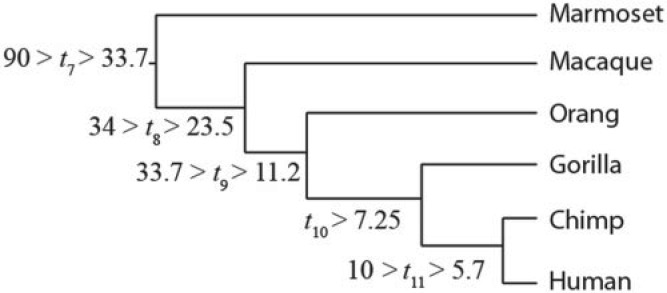
The phylogeny of six primate species with five fossil calibrations. The fossil bounds are soft, with 1% and 5% probabilities that minimum and maximum bounds are violated, respectively (Yang and Rannala 2006). The rationale for these fossil calibrations is given in Benton et al. (2009) and dos Reis et al. (2012).

We use both the old i.i.d. prior and the new Dirichlet prior for locus rates to estimate the divergence times on the six-species primate phylogeny studied by dos Reis and Yang (2013). The phylogeny with fossil calibrations is given in Figure 4. We use soft-bound fossil calibrations constructed following Benton et al. (2009) and dos Reis et al. (2012). The data are a subset of the large alignment analyzed by dos Reis et al. (2012), with 9992 protein-coding genes after ambiguous codons or alignment gaps were removed. We used the third codon positions only, and sampled loci with *N* ≥ 200 codons (7947 genes) randomly without replacement, to generate data sets of *L* = 1,2,10, and 300 loci. We generated 100 replicates for each *L*. Divergence times were then estimated using MCMCtree. The birth–death process parameters are λ = μ_BD_ = 1,ρ = 0. The time unit is 100 Myr. We use the independent-rates model and calculate the likelihood exactly under the HKY + G_5_ substitution model (Hasegawa et al. 1985; Yang 1994). For the old i.i.d. rate prior, three different priors for the locus rate are used: μ_*i*_∼*G*(2,2), μ_*i*_∼*G*(2,20), and μ_*i*_∼*G*(2,200), which have means 1, 0.1, and 0.01 corresponding to 10^−8^, 10^−9^, and 10^−10^ substitutions per site per year. The first prior rate is too fast and the last too slow. For the new Dirichlet prior implementation, three priors on the mean locus rate are used, 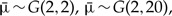 and 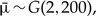 with α = 1.

The estimated divergence times using the old i.i.d. prior are shown in Table 3. The posterior time estimates are sensitive to the rate prior, in particular for the large number of loci, *L* = 300. Furthermore, the posterior rate estimates vary for the three rate priors. For example, with *L* = 300 and the fast rate prior, μ_*i*_∼*G*(2,2), the posterior mean of *t*_7_ (the age of crown Anthropoids) is 32.9 Ma (29.5, 35.5 Ma). These ages are too young and the posterior mean is very close to the 33.7 Ma minimum fossil bound applied to this node (Fig. 4). In fact, all posterior time estimates are too young and the minimum fossil bounds are violated for all nodes except node 7 (Fig. 4 and Table 3). Furthermore, the posterior mean rate is 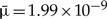 site^−1^ year^−1^ (1.84, 2.22), about twice the∼10^−9^ site^−1^ year^−1^ rate generally accepted for third codon positions in Primates. In contrast, with *L* = 300 and the slow-rate prior, μ_*i*_∼*G*(2,200), the posterior mean of *t*_7_ is 308.8 Ma (292.4, 325.8 Ma), much older than the 90 Ma maximum bound applied to this node (Fig. 4) and much older than the oldest mammal fossil ever found. Similarly, the ages for all other nodes are much older than their corresponding maximum fossil bounds. The mean rate is 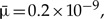 about five times less than the accepted rate of ∼10^−9^.

**Table 3.** Posterior means and 95% CIs of mean rate 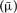 and divergence times (in Ma) among six primate species using the old i.i.d. prior

*L*	μ_*i*_∼	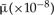	(95 %CI)	*t*_7_	(95 %CI)	*t*_8_	(95 %CI)	*t*_9_	(95 %CI)	*t*_10_	(95 %CI)	*t*_11_	(95 %CI)
*L* = 0	G(2,2)	1	(0.121, 2.786)	63.2	(34.8, 92.1)	29.7	(23.8, 34.6)	21.6	(12.0, 31.8)	12.9	(7.6, 23.3)	7.8	(5.8, 10.1)
	G(2,20)	0.1	(0.012, 0.271)	–	–	–	–	–	–	–	–	–	–
	G(2,200)	0.01	(0.001, 0.025)	–	–	–	–	–	–	–	–	–	–
*L* = 1	G(2,2)	0.113	(0.071, 0.172)	62.6	(39.1, 88.7)	30.0	(24.1, 34.6)	19.5	(12.8, 27.6)	10.3	(7.4, 15.4)	7.5	(5.8, 9.9)
	G(2,20)	0.101	(0.066, 0.15)	65.1	(40.8, 89.9)	30.4	(24.3, 34.7)	19.9	(13.1, 28)	10.5	(7.5, 15.8)	7.5	(5.8, 9.9)
	G(2,200)	0.058	(0.039, 0.082)	76.6	(51.2, 94.7)	31.7	(25.7, 35.1)	22.1	(14.7, 29.8)	11.5	(7.8, 17.7)	7.9	(5.8, 10.1)
*L* = 2	G(2,2)	0.110	(0.077, 0.153)	62.6	(42.1, 86)	30.1	(24.2, 34.6)	18.1	(12.8, 25)	9.7	(7.4, 13.4)	7.2	(5.7, 9.5)
	G(2,20)	0.098	(0.071, 0.133)	66.4	(45.2, 88.5)	30.7	(24.7, 34.8)	18.9	(13.3, 25.6)	10.0	(7.5, 13.9)	7.3	(5.8, 9.6)
	G(2,200)	0.057	(0.043, 0.072)	81.9	(62.2, 96.3)	32.7	(28, 35.6)	22.0	(16, 28.4)	11.5	(8.1, 16.3)	7.9	(5.9, 10)
*L* = 10	G(2,2)	0.120	(0.096, 0.148)	56.3	(44.3, 70.9)	28.6	(24, 33.8)	16.1	(12.9, 20)	8.1	(7.1, 9.8)	6.3	(5.7, 7.5)
	G(2,20)	0.097	(0.081, 0.117)	66.1	(52.8, 80.7)	31.9	(26.9, 35.1)	18.2	(14.6, 22.1)	8.8	(7.4, 10.9)	6.7	(5.7, 8.3)
	G(2,200)	0.051	(0.044, 0.058)	92.5	(82.1, 105.5)	37.1	(33.5, 42.2)	24.3	(20.4, 28.8)	12.2	(9.7, 14.9)	8.7	(6.8, 10.2)
*L* = 300	G(2,2)	0.199	(0.184, 0.222)	32.9	(29.5, 35.5)	17.2	(15.4, 18.7)	9.2	(8.2, 10)	4.2	(3.8, 4.6)	3.3	(2.9, 3.6)
	G(2,20)	0.098	(0.092, 0.103)	64.4	(60.6, 68.3)	32.5	(30.7, 34.3)	17.5	(16.5, 18.6)	7.8	(7.4, 8.3)	5.9	(5.6, 6.3)
	G(2,200)	0.019	(0.018, 0.02)	308.8	(292.4, 325.8)	150.4	(142.6, 158.5)	81.8	(77.3, 86.5)	36.3	(34.1, 38.6)	26.4	(24.7, 28.2)

Notes: Three priors for locus rates are used. *L* is the number of loci sampled from genomic data of protein-coding genes (with only 3rd codon positions used). The results for *L* = 0 correspond to the prior. The mean rate 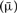 is calculated by averaging locus rates over loci from the MCMC samples. The results are averages of 100 replicates.

The estimated times using the new Dirichlet prior are shown in Table 4. In this case the posterior time estimates are rather insensitive to the rate prior, and the posterior of the average rate 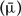 for all cases are very similar. For *L* = 300 loci, the posterior of the mean rate is 0.96 × 10^−9^ substitutions per site per year for all three rate priors. The new rate prior is clearly much better than the old one.

**Table 4.** Posterior means and 95% CIs of mean rate 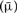 and divergence times (in Ma) among six primate species using the new Dirichlet prior

*L*	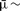	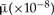	(95 %CI)	*t*_7_	(95 %CI)	*t*_8_	(95 %CI)	*t*_9_	(95 %CI)	*t*_10_	(95 %CI)	*t*_11_	(95 %CI)
*L* = 0	G(2,2)	1	(0.121, 2.786)	63.2	(34.8, 92.1)	29.7	(23.8, 34.6)	21.6	(12.0, 31.8)	12.9	(7.6, 23.3)	7.8	(5.8, 10.1)
	G(2,20)	0.1	(0.012, 0.271)	–	–	–	–	–	–	–	–	–	–
	G(2,200)	0.01	(0.001, 0.025)	–	–	–	–	–	–	–	–	–	–
*L* = 1	G(2,2)	0.113	(0.072, 0.172)	62.4	(39, 88.6)	30.1	(24.1, 34.6)	19.4	(12.8, 27.5)	10.3	(7.4, 15.4)	7.5	(5.8, 9.9)
	G(2,20)	0.101	(0.066, 0.15)	65.1	(40.8, 89.9)	30.4	(24.3, 34.7)	19.9	(13.1, 28)	10.5	(7.5, 15.8)	7.5	(5.8, 9.9)
	G(2,200)	0.058	(0.039, 0.082)	76.6	(51.2, 94.6)	31.7	(25.7, 35.1)	22.1	(14.7, 29.7)	11.5	(7.8, 17.7)	7.9	(5.8, 10.1)
*L* = 2	G(2,2)	0.110	(0.077, 0.153)	62.6	(42, 86)	30.1	(24.2, 34.6)	18.1	(12.8, 24.9)	9.7	(7.4, 13.4)	7.2	(5.7, 9.5)
	G(2,20)	0.098	(0.071, 0.133)	66.4	(45.2, 88.5)	30.7	(24.7, 34.8)	18.9	(13.3, 25.6)	9.9	(7.5, 13.9)	7.3	(5.8, 9.6)
	G(2,200)	0.057	(0.043, 0.072)	81.9	(62.3, 96.3)	32.7	(28, 35.6)	22.0	(16, 28.5)	11.5	(8.1, 16.4)	7.9	(5.9, 10.1)
*L* = 10	G(2,2)	0.120	(0.082, 0.125)	65.6	(51.6, 80.6)	31.7	(26.3, 35.1)	18.1	(14.4, 22)	9.0	(7.3, 10.9)	7.0	(5.7, 8.3)
	G(2,20)	0.097	(0.08, 0.121)	66.6	(52.7, 81.5)	31.9	(26.7, 35.2)	18.3	(14.6, 22.2)	9.0	(7.4, 11)	7.0	(5.7, 8.3)
	G(2,200)	0.051	(0.044, 0.058)	92.5	(82, 105.5)	37.1	(33.5, 42.2)	24.3	(20.4, 28.7)	12.0	(9.7, 14.9)	9.0	(6.8, 10.2)
*L* = 300	G(2,2)	0.096	(0.09, 0.103)	65.7	(60.9, 70.5)	33.1	(30.8, 35.3)	17.8	(16.5, 19.1)	8.0	(7.4, 8.6)	6.0	(5.7, 6.5)
	G(2,20)	0.096	(0.09, 0.103)	65.8	(61, 70.6)	33.1	(30.8, 35.3)	17.8	(16.6, 19.1)	8.0	(7.4, 8.6)	6.0	(5.7, 6.5)
	G(2,200)	0.096	(0.09, 0.103)	65.8	(61, 70.6)	33.1	(30.8, 35.3)	17.8	(16.6, 19.1)	8.0	(7.4, 8.6)	6.0	(5.7, 6.5)

Note: See note for Table 3.

## Divergence Time of Mammals

**Figure 5.**
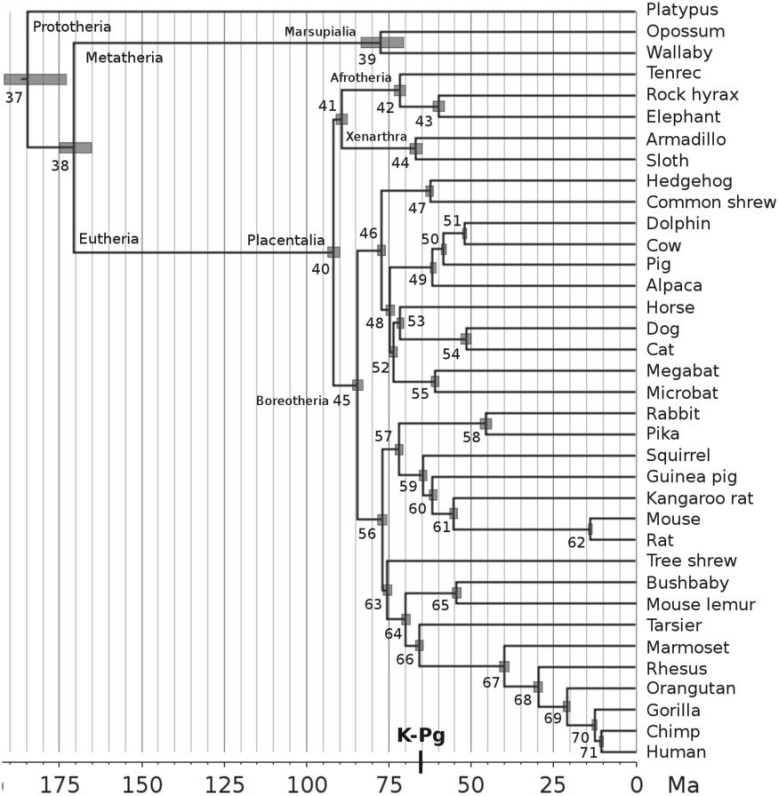
Timetree of mammals. Posterior divergence times were estimated using the new Dirichlet prior with 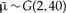 and α = 1. This prior has mean rate 0.05 × 10^−8^substitutions/site/year. Gray bars are 95% CIs. Nodes are numbered as in Table 5. The fossil calibrations are explained in detail in dos Reis et al. (2012). We summarize them here (in Ma, clade names refer to the crown groups): (37) Mammalia, min. 162.9, max. 191.1. (38) Theria, min. 124, max. 171.2. (39) Marsupialia, min. 48.6. (40) Placentalia, max. 131.5. (43) Paenungulata, min. 55.6. (44) Xenarthra, min. 55.6. (47) Eulipotyphla, min. 61.5. (49) Cetartiodactyla, max. 65.8. (51) Dolphin/Cow, min. 52.4. (53) Horse/cat, min. 62.5. (54) Carnivores, min. 39.68, max. 65.8. (55) Chiroptera, min. 48.6. (57) Glires, min. 61.5. (58) Lagomorpha, min. 48.6, max. 65.8. (59) Rodentia, min. 55.6, max. 65.8. (60) Guinea pig/rat, min. 52.8, max. 58.9. (61) Kangaroo rat/rat, min. 40.2, max. 56.0. (62) Muridae, min. 10.4, max. 14.0. (63) Euarchonta, min. 61.5. (64) Primates, min. 55.6. (65) Strepsirrhini, min. 33.7, max. 55.6. (67) Anthropoidea, min. 33.7. (68) Catarrhini, min. 23.5. (69) Hominidae, min. 11.2, max. 33.7. (70) Homininae, min. 7.25. (71) Hominini, min. 5.7, max. 10.0. All bounds are soft with 0.1% and 2.5% probabilities for left (min.) and right (max.) tails, respectively (Yang and Rannala 2006).

dos Reis et al. (2012) (see also dos Reis et al. 2014) estimated the divergence times of mammals using a data set of 36 mammal genomes (see Fig. 5 for the phylogeny and fossil calibrations). The data consists of a large alignment (∼21 million base pairs) of the 1st and 2nd codon positions sampled from ∼14,000 genes, and divided into 20 partitions. They used 26 fossil calibrations and a diffuse prior on the mean rate per partition, μ_*i*_∼*G*(1,1), with a time unit of 100 Myr. This rate prior has a mean of 1, meaning 10^−8^ substitutions per site per year. The parameters in the prior were chosen to give a large variance (and thus a diffuse prior), but the mean rate was too high: note that the estimated rate for even the third codon positions of those primate genes was only 0.96 × 10^−9^ sites^−1^ year^−1^ (Table 4). dos Reis et al. (2012) obtained very precise divergence time estimates (Table 5) that pointed to a diversification of modern placental mammals after the Cretaceous–Paleogene extinction event 66 Ma. Concerned that the i.i.d. rate prior with a high mean on 20 loci (partitions) may have had an undue influence on posterior time estimates, we repeat the analysis here using the new Dirichlet prior. Other aspects of the analysis are identical to those of dos Reis et al. (2012). The likelihood is calculated approximately (dos Reis and Yang 2011). We use two priors, 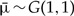 and 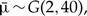 both with α = 1. The first prior has a mean rate that is too high (10^−8^ site^−1^ year^−1^) and the second a mean rate that is 20 times smaller (0.05 × 10^−8^), and appears to be more reasonable.

**Table 5.** Posterior estimates of divergence times of mammals (Ma)

		Old i.i.d. prior G(1,1)^*a*^		New Dirichlet G(1,1)		New Dirichlet G(2,40)	
Node	Crown clade	Mean	(95% CI)	Mean	(95% CI)	Mean	(95% CI)
37	Root	185.0	(174.5, 191.8)	185.7	(174.8, 191.9)	185.8	(174.4, 192.1)
38	Therian	175.4	(170.4, 181.7)	170.5	(165.1, 175.1)	170.2	(164.7, 175.1)
39	Marsupialia	66.7	(50.7, 83.7)	78.3	(69.9, 85.1)	77.7	(68.2, 84.8)
40	Placentalia	89.9	(88.3, 91.6)	91.7	(90, 93.4)	91.8	(89.9, 93.5)
41	Afrotheria/Xenartha	87.5	(85.9, 89.1)	89.3	(87.7, 91)	89.4	(87.6, 91.1)
42	Afrotheria	70.4	(68.5, 72.4)	71.6	(70.1, 73.1)	71.6	(70, 73.2)
43	Paenungulate	59.8	(57.7, 61.8)	59.9	(58.1, 61.5)	59.8	(58, 61.5)
44	Xenartha	70.0	(67.3, 72.4)	66.8	(65.1, 68.6)	66.8	(65, 68.7)
45	Boreotheria	82.4	(81.1, 83.8)	84.5	(83, 85.9)	84.5	(83, 86)
46	Laurasiatheria	76.0	(74.8, 77.1)	77.3	(76, 78.5)	77.3	(76, 78.6)
47	Lipotyphlan	61.3	(60.6, 61.8)	62.5	(61.5, 63.7)	62.5	(61.5, 63.8)
48	Cow/Alpaca	73.1	(72, 74.2)	74.6	(73.5, 75.8)	74.7	(73.4, 75.9)
49	Cetartiodactyla	61.4	(60.7, 62.3)	61.7	(60.8, 62.4)	61.7	(60.8, 62.5)
50	Pig/cow	58.0	(57.4, 58.8)	58.5	(57.6, 59.1)	58.5	(57.6, 59.2)
51	Dolphin/Cow	52.7	(52.2, 53.7)	52.2	(51.3, 52.6)	52.2	(51.4, 52.6)
52	Horse/cat/bat	72.2	(71.2, 73.3)	73.6	(72.5, 74.8)	73.7	(72.5, 74.9)
53	Horse/cat	70.1	(69.1, 71.1)	71.5	(70.4, 72.6)	71.5	(70.4, 72.7)
54	Carnivora	54.1	(52, 55.9)	51.5	(49.9, 53)	51.5	(50, 53.1)
55	Chiroptera	59.3	(57.6, 60.8)	61.0	(59.8, 62.2)	61.0	(59.8, 62.2)
56	Euarchontoglires	75.8	(74.6, 77)	77.1	(75.8, 78.3)	77.1	(75.7, 78.4)
57	Glires	70.7	(69.6, 71.8)	72.0	(70.8, 73.1)	72.0	(70.7, 73.2)
58	Lagomorpha	47.8	(45.8, 49.3)	45.5	(43.8, 47.2)	45.5	(43.8, 47.1)
59	Rodentia	64.5	(63.4, 65.5)	64.7	(63.6, 65.7)	64.7	(63.5, 65.7)
60	Guinea pig/rat	61.3	(60.3, 62.2)	61.7	(60.5, 62.6)	61.7	(60.5, 62.7)
61	Kangaroo rat/rat	55.6	(54.4, 56.5)	55.4	(54.2, 56.3)	55.4	(54.2, 56.3)
62	Mouse/rat	13.9	(13.2, 14.3)	14.0	(13.4, 14.4)	13.9	(13.4, 14.4)
63	Euarchonta	74.2	(73, 75.3)	75.4	(74.2, 76.6)	75.5	(74.1, 76.7)
64	Primates	69.0	(67.8, 70.1)	69.9	(68.7, 71)	69.9	(68.6, 71.1)
65	Strepsirrhini	54.3	(52.3, 55.8)	54.5	(53.1, 55.8)	54.5	(53.1, 55.8)
66	Human/tarsier	65.0	(63.9, 66)	65.8	(64.6, 66.9)	65.8	(64.6, 67)
67	Anthropoidea	36.6	(34.9, 38.3)	40.0	(38.5, 41.6)	39.9	(38.5, 41.3)
68	Catarrhini	25.6	(24.4, 26.8)	29.6	(28.4, 31.1)	29.6	(28.5, 30.8)
69	Human/orangutan	17.3	(16.2, 18.4)	21.0	(20.1, 22.1)	21.0	(20.1, 22)
70	Human/gorilla	10.2	(9.6, 11)	12.4	(11.8, 13.2)	12.4	(11.9, 13.1)
71	Human/chimp	8.7	(8.1, 9.4)	10.4	(9.9, 11.1)	10.4	(9.9, 11)

^*a*^Values from dos Reis et al. (2012). Note: Node numbers refer to those of Figure 5.

**Figure 6.**
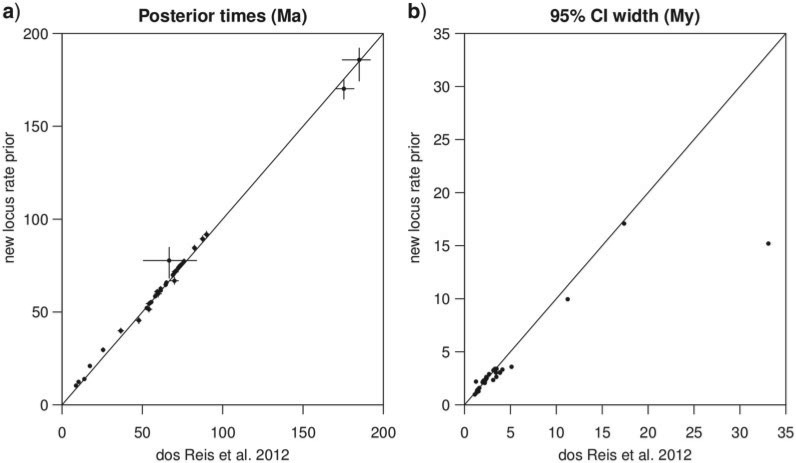
Divergence times of mammals. (A) The posterior means of times from dos Reis et al. (2012) are plotted against the values obtained here using the new Dirichlet prior, 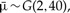 with α = 1. The vertical and horizontal bars indicate the 95% CI. (B) The posterior CI width of times from dos Reis et al. (2012) are plotted against the posterior CI width obtained using the new Dirichlet prior, as in (A). In both panels, the diagonal line is *y* = *x*.

Table 5 shows the results. Time estimates are relatively insensitive to the rate prior, and the results using the new Dirichlet prior are very similar to those using the old i.i.d. prior (Fig. 6a). Surprisingly, time estimates under the new prior tend to be more precise than those under the old i.i.d. prior (Fig. 6b). This trend is opposite to what we expected. We speculate that the large number of fossil calibrations on the mammal tree may have alleviated the impact of the misspecified rate prior on the posterior distribution of times. The timetree is shown in Figure 5. In accordance with previous results (dos Reis et al. 2012, 2014) we find that Placentalia originated in the Cretaceous before the K-Pg extinction 66 Ma, but the majority of crown placental orders originated in the Paleogene after the extinction (see also Meredith et al. 2011).

## Discussion

In Bayesian dating analysis, specification of the prior on divergence times is well recognized to be a complicated process, especially as the time prior incorporates fossil calibrations. As a result, much attention has been paid to the construction of the time prior (Kishino et al. 2001; Yang and Rannala 2006; Inoue et al. 2010; Heled and Drummond 2012). In contrast, less attention has been paid to the rate prior, perhaps because specification of the rate prior seems straightforward and the i.i.d. prior used in current computer programs appears to be quite innocent. However, times and rates are confounded parameters in the likelihood function, and as a result of the lack of identifiability, the priors for both sets of parameters will remain important even if an infinite amount of sequence data is available. This is quite unlike conventional Baysian inference, where priors become unimportant as more and more data is analyzed. Seemingly diffuse priors on the locus rates such as an exponential distribution with a large variance can have an unexpectedly strong effect on posterior time estimates. In this regard improper priors on the rates, available in some dating programs, may be the worst and should not be used.

The i.i.d. prior on locus rates makes an increasingly strong statement about the average locus rate with the increase of the number of loci, leading to very precise and over-confident posterior estimates when a large number of loci is included in the data. If the rate prior is unreasonable, the time estimates will be wrong with very narrow intervals. Although large uncertainty in posterior time estimates may not be desirable, the reduced uncertainty in time estimates caused by the i.i.d. prior is misleading, as the uncertainties associated with the fossil calibrations should remain even with many loci. Furthermore, in real data analysis it is impossible to predict the true rate, and the rate prior will always be misspecified to some extent, so that default priors that do not have an undue influence on the posterior may have a merit. Our study of the infinite-sites case as well as analysis of simulated and real data suggest that the new Dirichlet prior may circumvent both problems of false precision and undue prior influence associated with the current i.i.d. prior.

The extremely strong negative correlation between times and rates suggests that ideally one should specify the prior for times and rates jointly. However, specification of such a joint prior appears extremely difficult. Indeed our knowledge of the absolute rate appears to depend critically on our assumptions about the absolute times or interpretations of the fossil record. For example, despite the fact that the sequencing of the human and chimpanzee genomes has led to extremely precise estimates of the sequence divergence between the two species (1.3%; see, e.g., Burgess and Yang 2008), resolving this distance into absolute time and rate remains elusive (Scally and Durbin 2012). In this article, we have the less ambitious goal of constructing a rate prior that does not have a great impact on the posterior time estimates.

Users of dating programs other than MCMCtree v4.8 (which now implements the new Dirichlet rate prior) can use the following approach to construct an i.i.d. locus-rate prior that appears robust to rate prior misspecification and that avoids a decrease of prior uncertainty with the increase of the number of loci or site partitions (*L*). First, note that when α = α_μ_/*L*, equation (5) reduces to the density of *L* independent gamma variables, with μ_*i*_∼*G*(α_μ_/*L*,β_μ_/*L*). Then the μ_*i*_ have mean *m* = α_μ_/β_μ_ and variance 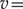

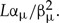
 Therefore, one may specify a gamma prior on μ_*i*_ with shape parameter α_μ_/*L* and rate parameter β_μ_/*L* (or scale parameter *s* = *L*/β_μ_). Even though this i.i.d. prior does not have the flexibility of the Dirichlet prior implemented in this article (for example, α in equation 5 is always fixed at α_μ_/*L* in the i.i.d. prior) and its specification depends on the number of loci in the data set being analyzed, our preliminary test suggests that it may produce similar time estimates to the Dirichlet prior when the number of loci is not very large.

Finally, we note that there are other sources of errors or uncertainties involved in divergence time estimation that are not dealt with in this study. Foremost is the difficulties with the interpretation of the fossil record to formulate calibrations in a molecular clock dating analysis. For example, one never really knows the difference between the age of a fossil and the age of the node that is being calibrated by the fossil, and even the placement of the fossil on the phylogeny may also be uncertain. In this article, we do not explicitly deal with such factors that affect the quality of fossil calibrations but assume that the fossil bounds or calibration densities adequately summarize the information and uncertainties in the fossil record. Furthermore, gene genealogies at individual loci or genomic regions may differ from the species tree due to polymorphism in ancestral species, and the coalescent times of lineages within a locus may be older than the time of divergence between species (Burgess and Yang 2008). This source of uncertainty may not be important when ancient divergences are studied, but it can be considerable when divergence times between closely related species such as human and the chimpanzee are estimated. For example, at about 70% of loci, human and chimpanzee are more closely related to each other than each is to gorilla (Burgess and Yang 2008; Scally et al. 2012), but at the remaining loci the (true) gene tree differs from the species tree. This source of uncertainty is ignored in our study here.

## Funding

This work was supported by Biotechnology and Biological Sciences Research Council (BBSRC), UK, grant BB/J009709/1. Z.Y. is a Royal Society Wolfson Merit award holder. T.Z. is supported by Natural Science Foundation of China (NSF) grants (31301093, 11301294 and 11201224).

## References

[B1] Benton M., Donoghue P., Asher R., Hedges B.S., Kumar S. (2009). Calibrating and constraining molecular clocks. The Timetree of Life.

[B2] Burgess R., Yang Z. (2008). Estimation of hominoid ancestral population sizes under bayesian coalescent models incorporating mutation rate variation and sequencing errors. Mol. Biol. Evol.

[B3] dos Reis M., Donoghue P.C., Yang Z. (2014). Neither phylogenomic nor palaeontological data support a Palaeogene origin of placental mammals. Biol. Lett..

[B4] dos Reis M., Inoue J., Hasegawa M., Asher R.J., Donoghue P.C., Yang Z. (2012). Phylogenomic datasets provide both precision and accuracy in estimating the timescale of placental mammal phylogeny. Proc. Biol. Sci..

[B5] dos Reis M., Yang Z. (2011). Approximate likelihood calculation on a phylogeny for Bayesian estimation of divergence times. Mol. Biol. Evol..

[B6] dos Reis M., Yang Z. (2013). The unbearable uncertainty of Bayesian divergence time estimation. J. Syst. Evol..

[B7] Drummond A.J., Ho S.Y., Phillips M.J., Rambaut A. (2006). Relaxed phylogenetics and dating with confidence. PLoS Biol..

[B8] Drummond A.J., Suchard M.A., Xie D., Rambaut A. (2012). Bayesian phylogenetics with BEAUti and the BEAST 1.7. Mol. Biol. Evol..

[B9] Hasegawa M., Kishino H., Yano T. (1985). Dating of the human-ape splitting by a molecular clock of mitochondrial DNA. J. Mol. Evol..

[B10] Heath T.A., Holder M.T., Huelsenbeck J.P. (2012). A dirichlet process prior for estimating lineage-specific substitution rates. Mol. Biol. Evol..

[B11] Heled J., Drummond A. (2012). Calibrated trees priors for relaxed phylogenetics and divergence time estimation. Syst. Biol..

[B12] Huelsenbeck J.P., Larget B., Swofford D. (2000). A compound poisson process for relaxing the molecular clock. Genetics.

[B13] Inoue J., Donoghue P.C., Yang Z. (2010). The impact of the representation of fossil calibrations on Bayesian estimation of species divergence times. Syst. Biol..

[B14] Jukes T.H., Cantor C.R., Munro H.N. (1969). Evolution of protein molecules. Mammalian protein metabolism.

[B15] Kimura M. (1969). The number of heterozygous nucleotide sites maintained in a finite population due to steady flux of mutations. Genetics.

[B16] Kimura M. (1983). The neutral theory of molecular evolution.

[B17] Kishino H., Thorne J., Bruno W. (2001). Performance of a divergence time estimation method under a probabilistic model of rate evolution. Mol. Biol. Evol..

[B18] Lepage T., Bryant D., Philippe H., Lartillot N. (2007). A general comparison of relaxed molecular clock models. Mol. Biol. Evol..

[B19] Meredith R.W., Janecka J.E., Gatesy J., Ryder O.A., Fisher C.A., Teeling E.C., Goodbla A., Eizirik E., Simao T.L., Stadler T., Rabosky D.L., Honeycutt R.L., Flynn J.J., Ingram C.M., Steiner C., Williams T.L., Robinson T.J., Burk-Herrick A., Westerman M., Ayoub N.A., Springer M.S., Murphy W.J. (2011). Impacts of the cretaceous terrestrial revolution and KPg extinction on mammal diversification. Science.

[B20] Rannala B., Yang Z. (2007). Inferring speciation times under an episodic molecular clock. Syst. Biol..

[B21] Rannala B., Zhu T., Yang Z. (2012). Tail paradox, partial identifiability, and influential priors in Bayesian branch length inference. Mol. Biol. Evol..

[B22] Ronquist F., Teslenko M., van der Mark P., Ayres D.L., Darling A., Hohna S., Larget B., Liu L., Suchard M.A., Huelsenbeck J.P. (2012). MrBayes 3.2: efficient Bayesian phylogenetic inference and model choice across a large model space. Syst. Biol..

[B23] Scally A., Durbin R. (2012). Revising the human mutation rate: implications for understanding human evolution. Nat. Rev. Genet..

[B24] Scally A., Dutheil J.Y., Hillier L.W., Jordan G.E., Goodhead I., Herrero J., Hobolth A., Lappalainen T., Mailund T., Marques-Bonet T., McCarthy S., Montgomery S.H., Schwalie P.C., Tang Y.A., Ward M.C., Xue Y., Yngvadottir B., Alkan C., Andersen L.N., Ayub Q., Ball E.V., Beal K., Bradley B.J., Chen Y., Clee C.M., Fitzgerald S., Graves T.A., Gu Y., Heath P., Heger A., Karakoc E., Kolb-Kokocinski A., Laird G.K., Lunter G., Meader S., Mort M., Mullikin J.C., Munch K., O'Connor T.D., Phillips A.D., Prado-Martinez J., Rogers A.S., Sajjadian S., Schmidt D., Shaw K., Simpson J.T., Stenson P.D., Turner D.J., Vigilant L., Vilella A.J., Whitener W., Zhu B., Cooper D.N., de Jong P., Dermitzakis E.T., Eichler E.E., Flicek P., Goldman N., Mundy N.I., Ning Z., Odom D.T., Ponting C.P., Quail M.A., Ryder O.A., Searle S.M., Warren W.C., Wilson R.K., Schierup M.H., Rogers J., Tyler-Smith C., Durbin R. (2012). Insights into hominid evolution from the gorilla genome sequence. Nature.

[B25] Thorne J.L., Kishino H, Painter I.S. (1998). Estimating the rate of evolution of the rate of molecular evolution. Mol. Biol. Evol..

[B26] Yang Z. (1994). Maximum likelihood phylogenetic estimation from DNA sequences with variable rates over sites: approximate methods. J. Mol. Evol..

[B27] Yang Z. (2007). PAML 4: phylogenetic analysis by maximum likelihood. Mol. Biol. Evol..

[B28] Yang Z., Rannala B. (2006). Bayesian estimation of species divergence times under a molecular clock using multiple fossil calibrations with soft bounds. Mol. Biol. Evol..

[B29] Zhang C., Rannala B., Yang Z. (2012). Robustness of compound Dirichlet priors for Bayesian inference of branch lengths. Syst. Biol..

